# Comparison of Anxiety Using the Amsterdam Preoperative Anxiety and Information Scale Score in Preoperative Patients Counselled Through Verbal Versus Multimedia Mode

**DOI:** 10.7759/cureus.84622

**Published:** 2025-05-22

**Authors:** Nikhil Shukla, Pooja Trivedi, Neha Dubey, Abhiram A Awasthi

**Affiliations:** 1 Department of Anesthesiology, People's College of Medical Sciences & Research Centre, Bhopal, IND; 2 Department of Anesthesiology, Amaltas Institute of Medical Sciences, Dewas, IND; 3 Department of Orthopedics and Traumatology, Datta Meghe Institute of Higher Education and Research, Wardha, IND

**Keywords:** amsterdam preoperative anxiety and information scale (apais), anesthesia, apais score, multimedia counseling, preoperative anxiety, verbal counseling

## Abstract

Background

Preoperative anxiety is common and can affect surgical outcomes. This study compares the effectiveness of verbal and multimedia counseling in reducing preoperative anxiety, measured using the Amsterdam Preoperative Anxiety and Information Scale (APAIS). Due to the lower literacy rate in India, it is difficult for patients to read, understand, and retain information. Therefore, there was a need to compare various modes of counseling.

Objective

The aim of this study was to determine if multimedia counseling is more effective than verbal counseling in reducing preoperative anxiety in patients scheduled for elective surgery.

Methods

A randomized controlled trial was conducted with 90 adult patients scheduled for elective surgery. Participants were randomly assigned to receive either verbal counseling (group A) or multimedia counseling (group B). Anxiety levels were assessed before and after counseling using the APAIS score. Statistical analysis was performed to compare changes in anxiety levels between the two groups.

Results

Both groups showed reduced anxiety after counseling, but the multimedia group had a significantly greater reduction in APAIS scores compared to the verbal counseling group (p < 0.001). The multimedia group also reported higher satisfaction with the counseling process.

Conclusion

Multimedia counseling significantly reduces preoperative anxiety more effectively than verbal counseling. This approach could be particularly beneficial in settings where patients may have lower literacy levels.

## Introduction

An uncomfortable feeling that jeopardizes a patient's comfort and health is anxiety [1]. The term "preoperative anxiety" describes the uneasiness, fear, or nervousness people feel before undergoing surgery. Fear of the unknown, anxieties about the surgical outcome, discomfort or complications, and being apart from loved ones during the surgery are some of the causes of this anxiety. By using a variety of techniques and encouraging improved surgical experiences and results, healthcare providers can reduce preoperative anxiety [2].

Accurately assessing preoperative anxiety levels is challenging yet imperative for optimizing patient care. Various validated questionnaires, including the Amsterdam Preoperative Anxiety and Information Scale (APAIS) and the visual analog scale, are utilized to gauge anxiety levels in patients [3,4]. Of these, APAIS is widely acknowledged for evaluating anxiety related to anesthesia, surgery, and patients' desire for information. In 1996, the APAIS was created to measure patients' preoperative anxiety [5]. The APAIS has been tested in various countries and used for the optimization of modes of counselling all over the world for producing better intraoperative and postoperative outcomes. Since the Indian population has a lower literacy rate, it is difficult for patients and their attendants to understand complicated anesthetic procedures verbally; thus, multimedia, such as audiovisuals depicting anesthetic procedures, becomes a handy tool [4,6,7].

To reduce anxiety in preoperative patients, the aim of this study is to compare verbal counselling with multimedia counseling using the APAIS score. It also sought to identify the causes of preoperative anxiety, including fear of death, fear of waking up during surgery, postoperative pain, postoperative nausea and vomiting, fear of needles, and fear of intervention.

## Materials and methods

This prospective randomized comparative study was conducted from September 1, 2024, to November 30, 2024, at People’s College of Medical Science and Research Centre, Bhopal, Madhya Pradesh, India, after receiving approval from the institutional ethics committee (PCMS/OD/PS/2024/1794). All cases posted for elective surgeries during the study period in the Department of Anesthesia were included in the study. The inclusion criteria were American Society of Anesthesiologists (ASA) grade I, II, or III and patients aged 18 to 60 years of either gender. Study variables were the type of anesthesia, surgical history, and APAIS score. Exclusion criteria included patients not willing to give consent, those with psychiatric disorders, those visually challenged, those with a history of previous surgery or anesthetic exposure, and those with ASA grade IV. The materials used were an APAIS questionnaire sheet and a multimedia video of an anesthesiologist performing pre-anesthetic check-ups on a laptop or mobile phone.

Patients were divided into two groups randomly, with each group consisting of 45 patients.

Calculation of sample size

Previous studies, such as those by Jlala et al. [4] and Ruffinengo et al. [7], used sample sizes of 40-50 participants per group to detect significant differences in preoperative anxiety levels with comparable interventions.

Power calculation was conducted using an estimated medium effect size (Cohen’s d = 0.5) and aiming for 80% power at a 5% significance level. A sample size of 45 participants per group was calculated using the following formula:

$\frac{ n = 2 \times (Z_{&alpha;/2} + Z_{&beta;})^{2} \times \sigma^{2}}{ &Delta;^{2}}$

where *Z_α/2_* = 1.96 (for α = 0.05); *Z_β_* = 0.84 (for 1-β = 0.8), σ is the standard deviation of APAIS scores (based on previous studies or pilot data), and Δ is the minimum clinically meaningful difference in APAIS scores.

This yielded a required sample size of 40 per group, and we included 45 participants per group to account for potential dropouts or missing data.

Firstly, the APAIS score was monitored in both groups. One hour prior to surgery, group A was counseled verbally using the counseling script by a trained anesthesiologist. The counseling session covered the following aspects in detail:

Explanation of the anesthesia procedure: description of the type of anesthesia to be administered (e.g., general, regional, or local) and steps involved in the administration of anesthesia

Safety and monitoring: assurance about the safety measures taken during anesthesia and explanation of continuous monitoring during the procedure to ensure safety

Side effects and complications: common side effects such as nausea, drowsiness, and temporary disorientation, as well as rare, but possible, complications such as allergic reactions or prolonged sedation

Recovery and postoperative care: what to expect during recovery from anesthesia, as well as instructions for postoperative care

Addressing patient-specific concerns: any personal fears or misconceptions about anesthesia were discussed and addressed, and patients were encouraged to ask questions to clarify doubts and alleviate concerns.

Group B received counseling through a multimedia video, which demonstrated the anesthesia technique, available on YouTube, mostly.

For patients who understood only Hindi, Videos 1, 2 were used, while for patients who understood English as well, Video 3 was used. All videos were downloaded in advance to skip advertisements and for convenience.

**Video 1:** Spinal anesthesia procedure

**Video 2:** Spinal and epidural anesthesia

**Video 3:** Anesthesia sedation: what to expect

Anxiety level was calculated using an APAIS score by an independent person. Anxiety levels were classified as low (0-3), moderate (4-6), or high (7-10). The APAIS consists of six questions assessing both anesthesia- and surgery-related anxiety and the patient’s desire for information (Table 1).

**Table 1:** The Amsterdam Preoperative Anxiety and Information Scale [3] A five-point Likert scale, with 1 denoting not at all and 5 denoting highly, should be used to determine how much agreement one has with these assertions.

APAIS Question		
1.	I am worried about the anesthetic	(1) (2) (3) (4) (5)
2.	The anesthetic is on my mind continually	(1) (2) (3) (4) (5)
3.	I would like to know as much as possible about the anesthetic	(1) (2) (3) (4) (5)
4.	I am worried about the procedure	(1) (2) (3) (4) (5)
5.	The procedure is on my mind continually	(1) (2) (3) (4) (5)
6.	I would like to know as much as possible about the procedure	(1) (2) (3) (4) (5)

Each question is rated on a scale of 1 to 5, and total anxiety scores were categorized as low (4-8), moderate (9-15), and high (16-20). Information desire scores were also classified as low, moderate, and high.

Statistical analysis

All the data analyses were performed using SPSS Version 20 (IBM Corp., Armonk, NY). Frequency distribution and cross-tabulation were performed to prepare the tables. For comparing post-counseling data, an unpaired t-test was used. Level of significance was assessed at 5%.

## Results

A total of 90 patients were included in the study after meeting the eligibility criteria for three months. Among them, 45 were assigned to group B, which received counseling in multimedia form, and 45 to the control group A, which received verbal counseling only. All participants completed the study, and no participants were excluded at any stage. Every patient who participated in the study remained for the entire duration and was included in the final analysis.

The demographic characteristics, including average age, gender distribution, and ASA classification, were comparable between the control groups A and B, with no statistically significant differences (p > 0.05) (Table 2).

**Table 2:** Demographic profile No statistical difference is seen in demographic profile (p > 0.05), calculated using the Student unpaired t-test. ASA, American Society of Anesthesiologists; F, female; M, male

Groups	Group A	Group B	P-value
Mean age±standard deviation	42.82±3.64	43.15±3.67	>0.55
ASA I/II	11/34	15/30	
Sex distribution (M:F)	23:22	21:24	
Previous surgery (no/yes)	28/17	31/14	

The mean age was 42.82 years in the control group and 43.15 years in the multimedia group. There were no discernible variations in the distribution of genders or educational levels between the two groups (p > 0.05).

**Figure 1:**
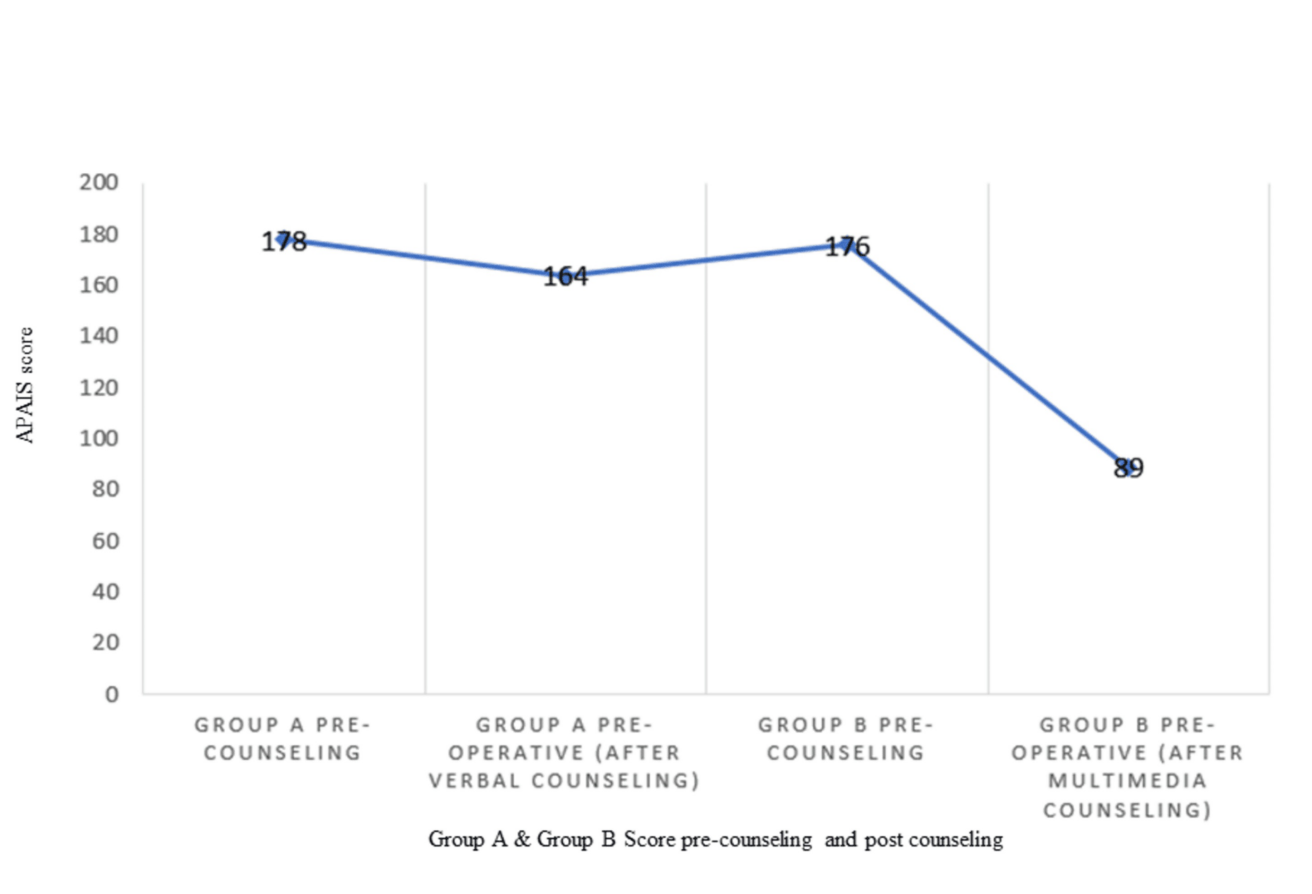
∑x of APAIS score in both groups APAIS, Amsterdam Preoperative Anxiety and Information Scale

The study revealed that both groups had similar anxiety levels pre-counseling when measured using the APAIS score. Group B experienced a more substantial reduction in the APAIS score, which was associated with greater patient satisfaction and comfort. At baseline, anesthesia-related anxiety was similar between group A and group B (p > 0.05). One hour before surgery, anxiety significantly decreased in both groups, with a more pronounced reduction seen in group B, with an F-ratio value of 78.95 and a p-value of <0.00001, as depicted in Table 3. The difference in ∑x of APAIS score in both groups before and after counseling (verbal and multimedia counseling, respectively) is shown in Figure 1.

**Table 3:** Comparison of heart rate at various intervals A p-value of >0.05 was considered insignificant, calculated using the Student unpaired t-test

Heart rate	Group A, Mean±Standard Deviation	Group B, Mean±Standard Deviation	T-score	P-value
Baseline	84.5±11.13	85.08±11.09	-0.25	0.80
Post-intervention	90.55±14.42	90.16±15.11	0.13	0.90
Immediate pre-operative	87.18±12.60	86.4±16.13	0.26	0.80

Mean heart rate was comparable between both groups at the baseline, post-intervention, and immediate preoperative (Table 3).

There was a significant increase in heart rate in both groups following intervention. However, this increase was less pronounced in the multimedia group. Immediate preoperative heart rate in the multimedia group was closer to the baseline level than in the verbal group.

## Discussion

Preoperative anxiety is a significant issue that patients face since it can result in emotional, mental, and physical issues [7,8]. It is described as "an unpleasant, restless, or tense state in which the patient is worried about illness, hospitalization, anesthesia, surgery, or the unknown." Given that more than 312.9 million surgeries are carried out annually worldwide, it is critical to properly gauge how each patient feels about the processes and results. Anxiety prior to surgery is thought to affect 25-80% of patients, which can result in hyperalgesia and delayed recovery [9,10]. All of the patients in our study experienced anxiety to varying degrees prior to surgery, and several of them experienced severe levels of anxiety. Anxiety can arise for a variety of reasons, including fear of surgery or anesthesia.

Previous studies conducted in Western countries, such as the one by Jlala et al. [4], have shown that patients' preoperative anxiety drastically reduced when they were shown a multimedia clip depicting procedures at the time of preoperative examination, leading to better perioperative hemodynamic control and a better postoperative outcome. This study reflects similar results in the Indian population as well. Celik and Edipoglu [11] found the APAIS score to be an important tool to assess preoperative anxiety; it was used effectively in this study to determine preoperative anxiety. Preoperative anxiety is a neglected problem that needs to be regularly evaluated at preoperative anesthesia checkups, and anesthesiologists should provide counseling to patients who are displaying elevated anxiety, according to Bansal and Joon [12]. There are several ways to quantify preoperative anxiety, and each has advantages and disadvantages. Some popular methods include self-report questionnaires, physiological measurements, observational scales, psychological interviews, and combination approaches [2].

Visual aids such as pictures combined with text have been demonstrated to significantly improve patient understanding, retention, and overall experience in medical counseling, especially regarding anesthesia. For many patients, visual learning is essential in demystifying complicated medical concepts and making procedures and possible side effects more approachable and understandable. By graphically representing what will happen, this method helps clarify medical procedures such as anesthesia and surgery, which lowers anxiety and misunderstanding. Additionally, compared to verbal counseling alone, visual aids and text work better together as memory aids, enhancing the recall of important information and involving patients more fully.

Verbal anesthesia counseling that incorporates text and images provides a thorough, interesting, and approachable communication method that enhances patient comprehension, lowers anxiety, and improves medical results.

The ASA physical status classification system mainly determines patients' preoperative anxiety levels. It is anticipated that people with higher ASA physical status categories, which is a sign of more complex medical conditions and comorbidities, will feel more anxious than people with lower ASA classifications. Increased anxiety is probably caused by a number of things, such as worries about pre-existing medical issues, uncertainty about the results of surgery, and a sense of losing control over the procedure. In order to effectively manage anxiety and enhance overall patient outcomes for patients with substantial medical comorbidities, it is imperative to comprehend these dynamics and customize perioperative care regimens accordingly.

Additionally, it was shown that the majority of patients who reported preoperative anxiety did so due to worry related to anesthesia and operation. These results align with those reported by Mackenzie [13], who discovered that anesthesia, surgery, or a combination were the primary sources of concern in 23%, 27%, and 38% of patients, respectively. Preoperative anxiety is a common occurrence before surgery and can have a variety of causes.

Patients' fear of the unknown is exacerbated by uncertainty about the surgical process and its results. The expectation of perioperative pain and worries about possible adverse anesthesia side effects, such as allergic responses and unconsciousness, exacerbate anxiety levels. Preoperative anxiety is exacerbated when people feel unprepared and alone due to a lack of information and support from social networks and healthcare professionals. It is imperative to identify and address these complex contributors for thorough preoperative treatment and to maximize patient experience and results [14,15].

Limitations

One notable limitation of the present study is the lack of examination of gender-based differences in anxiety responses. Given that anxiety can vary significantly between males and females, exploring gender-specific effects could have provided additional insights. The sample size was small and single-centered. Thus, the result should be verified by conducting studies with a larger cohort. Also, verbal counseling done by a counselor is subjective and can cause variation in results.

This research was intended as a preliminary exploration to assess feasibility, refine methods, and identify trends. Time constraints and logistical factors further restricted participant availability. The study adhered to ethical guidelines, avoiding over-recruitment when initial data could meet exploratory objectives.

Due to unforeseen recruitment challenges, the final sample size fell short, potentially reducing statistical power. Despite limitations, the results offer valuable insights, providing a foundation for future research. Trends observed in this study can inform the design and execution of larger-scale studies.

## Conclusions

This study reveals that using multimedia counseling to educate patients about anesthesia and anesthetics significantly lowers preoperative anxiety compared to solely verbal information. Patients who viewed the multimedia film experienced more substantial decreases in anxiety related to anesthesia, surgery, and procedural information. Additionally, the multimedia counseling approach received positive patient reviews, enhancing their overall perioperative experience. Therefore, incorporating audiovisual resources into preoperative education and counseling proves to be an effective strategy for reducing preoperative anxiety in patients scheduled for surgery.
